# Algorithmic Habituation: A Neurocognitive and Systems-Based Framework for Human–AI Co-Adaptation

**DOI:** 10.3390/brainsci16050473

**Published:** 2026-04-28

**Authors:** Narcisa Carmen Mladin, Dana Rad, Dumitru Ștefan Coman, Miron Gavril Popescu, Maria Iulia Felea, Radiana Marcu, Gavril Rad

**Affiliations:** 1 Department of Internal Medicine, “Vasile Goldis” Western University of Arad, B-dul Revolutiei Nr. 96, 310025 Arad, Romania; mladin_narcisa@yahoo.com; 2 Center of Research Development and Innovation in Psychology, Faculty of Educational Sciences Psychology and Social Work, Aurel Vlaicu University of Arad, 310025 Arad, Romania; radi.marcu@yahoo.com (R.M.); radgavrilarad@gmail.com (G.R.); 3 Faculty of Humanities and Social Sciences, Aurel Vlaicu University of Arad, 310025 Arad, Romania; stefeco@gmail.com (D.Ș.C.); popescu.mirongavril@gmail.com (M.G.P.); 4 Department for Teacher Training, “1 Decembrie 1918” University of Alba Iulia, 510009 Alba Iulia, Romania; felea.mariaiulia95@gmail.com

**Keywords:** algorithmic habituation, human–AI interaction, neuroplasticity, predictive processing, cognitive adaptation

## Abstract

**Highlights:**

**What are the main findings?**

- The study introduces algorithmic habituation as a novel cognitive process describing how users progressively adapt to the predictive regularities of AI systems.
- A mechanistic co-adaptation loop is proposed, alongside a four-dimensional typology (cognitive, decisional, creative, and moral habituation) explaining how AI reshapes human cognition and behavior.

**What are the implications of the main findings?**

- Algorithmic habituation may enhance efficiency but can also reduce critical thinking, increase automation bias, and standardize cognitive and behavioral patterns.
- The framework highlights the need for designing AI systems that preserve human agency, reflexivity, and ethical awareness in increasingly adaptive human–AI ecosystems.

**Abstract:**

Background/Objectives: As artificial intelligence systems become increasingly embedded in everyday cognitive tasks, human–AI interaction is no longer limited to tool use but evolves into a dynamic process of mutual adaptation. While extensive research has examined algorithmic learning, far less attention has been given to how users progressively adapt to AI systems. This paper introduces the concept of algorithmic habituation, defined as the gradual accommodation of users to the regularities and predictive patterns of AI systems. The objective is to provide a neurocognitive and systems-based framework that explains this phenomenon. Methods: The study develops a conceptual and integrative framework grounded in classical theories of habituation, neuroplasticity, predictive processing, and systems theory. Building on these foundations, we propose a mechanistic model of human–AI co-adaptation, conceptualized as a recursive feedback loop involving repeated interaction, pattern recognition, expectation stabilization, and cognitive economy. In addition, a typology of algorithmic habituation is advanced, alongside proposed empirical pathways for future validation, including scale development, experimental paradigms, and longitudinal designs. Results: The proposed framework suggests that repeated interaction with AI systems leads to stabilization of cognitive expectations, reduced cognitive effort, and increased behavioral standardization. This process extends beyond perceptual habituation into higher-order domains, including decision-making, creativity, and moral judgment. The typology identifies four primary forms of algorithmic habituation: cognitive, decisional, creative, and moral. The model predicts both adaptive outcomes (efficiency, reduced cognitive load) and maladaptive consequences (reduced reflexivity, automation bias, and potential erosion of critical thinking). Conclusions: Algorithmic habituation represents a novel construct at the intersection of neuroscience, cognitive psychology, and human–AI interaction. By framing user adaptation as a form of neurocognitively grounded habituation within recursive systems, this paper contributes a new perspective to understanding AI integration in human cognition. The framework has implications for digital wellbeing, education, and AI ethics, and opens multiple avenues for empirical research.

## 1. Introduction

### 1.1. From Tool Use to Cognitive Co-Adaptation

The rapid integration of artificial intelligence (AI) systems into everyday cognitive activities has fundamentally transformed the nature of human–technology interaction. In this context, AI systems can be broadly understood as computational systems capable of performing tasks that typically require human intelligence, including learning from data, pattern recognition, prediction, and adaptive decision-making. Traditionally, technological tools were conceptualized as passive instruments designed to extend human capabilities without significantly reshaping underlying cognitive processes. However, contemporary AI systems—particularly adaptive, interactive, and predictive architectures—operate within dynamic feedback loops that actively respond to user behavior, thereby contributing to the gradual reconfiguration of human cognition itself.

Early theoretical contributions highlighted that interaction with complex systems involves not only user adaptation to technology but also system adaptation to users, giving rise to processes of co-adaptation [1]. This perspective has been expanded in subsequent work demonstrating that human–machine interaction is inherently iterative, involving continuous mutual adjustments across cognitive and behavioral levels [2]. Rather than static usage, interaction becomes a process of reciprocal calibration, in which both agents evolve through repeated engagement.

Empirical studies in human–machine and human–robot interaction further support this view, showing that co-adaptation emerges through feedback-driven mechanisms such as error correction, expectation updating, and learning processes [3,4]. These dynamics are evident in neuro-adaptive systems, where physiological signals are used to adjust system behavior in real time [4], as well as in embodied interaction contexts, where teams of humans and intelligent agents develop shared patterns of coordination [5]. Longitudinal perspectives extend this understanding by suggesting that co-adaptation unfolds over time, especially in interactions involving everyday technologies and diverse user populations [6].

More recent frameworks emphasize that co-adaptation is not merely behavioral but also computational and systemic, involving multi-level interactions between cognitive processes, decision-making strategies, and adaptive algorithms [7]. Computational modeling approaches have further demonstrated that human learning and decision-making can be shaped through interaction with adaptive systems, reinforcing the recursive nature of co-adaptation [8]. Within this broader context, human–AI interaction can be understood as a co-evolutionary process, in which both user and system trajectories are continuously modified through interaction.

Emerging research on AI systems, particularly large language models, suggests that users progressively align their communication styles, expectations, and problem-solving strategies with algorithmic outputs [9]. This alignment is not trivial: it reflects deeper processes of cognitive restructuring, potentially leading to both enhancement and degradation effects, as highlighted by the “cognitive atrophy paradox,” which suggests that while AI systems can augment cognitive performance in the short term, prolonged reliance may reduce independent cognitive engagement and skill retention over time [10]. At the same time, advances in collaborative and knowledge-driven AI architectures reinforce the idea that human–AI systems are evolving toward tightly coupled, co-adaptive ecosystems [11]. Recent empirical findings further confirm that users actively adapt to AI systems in measurable ways, including linguistic convergence and interaction pattern stabilization [12,13].

Taken together, these developments indicate a fundamental shift: from viewing AI as a tool to understanding it as a cognitive partner within a co-adaptive system. However, while the concept of co-adaptation has been extensively explored, the specific mechanisms through which users progressively internalize and stabilize interaction patterns with AI systems remain insufficiently theorized.

### 1.2. The Missing Piece: User Adaptation to AI

Despite the growing body of literature on co-adaptive systems, the dominant focus has been placed on system design, algorithmic optimization, and performance outcomes. Comparatively little attention has been given to the user-side adaptation processes, particularly those that unfold gradually and implicitly over repeated interactions with AI systems.

Existing studies acknowledge that users adapt their behavior in response to system feedback, yet these processes are often conceptualized in terms of usability, efficiency, or task performance rather than deeper cognitive restructuring [2,7]. Even in more advanced models of co-adaptation, user adaptation is typically treated as a secondary effect of system learning rather than as a central phenomenon requiring independent theoretical articulation.

This gap becomes particularly salient in the context of contemporary AI systems, which are characterized by high levels of predictability, responsiveness, and personalization.

Recent discussions on human alignment with AI systems suggest that users may adapt more extensively than previously assumed, adjusting their communication strategies and expectations to better fit algorithmic behavior [12]. Similarly, studies on learner–AI interaction indicate that co-adaptation can occur at multiple linguistic and cognitive levels, reflecting deeper integration of AI into cognitive processes [13]. These findings point toward a critical but underexplored phenomenon: the progressive stabilization of user cognition in response to algorithmic regularities.

### 1.3. Defining Algorithmic Habituation

To address this gap, the present study introduces the concept of algorithmic habituation, defined as the gradual accommodation of users to the regularities, constraints, and predictive patterns of AI systems. To our knowledge, the concept of algorithmic habituation has not been formally defined in the literature. Unlike classical forms of habituation, which refer to diminished responses to repeated stimuli in biological systems, algorithmic habituation operates at a higher cognitive level, involving the internalization of interaction patterns within human–AI systems.

This process can be understood as emerging from repeated exposure to structured outputs, leading to the formation of expectations, reduction in cognitive effort, and stabilization of behavioral responses. In this sense, algorithmic habituation represents a cognitive–algorithmic analogue of habituation, extending the concept from sensory processing to complex, technology-mediated cognition.

Importantly, algorithmic habituation is not inherently negative. On the one hand, it may enhance efficiency, reduce cognitive load, and facilitate smoother interaction with AI systems. On the other hand, it may contribute to reduced reflexivity, over-reliance on algorithmic outputs, and the emergence of automation bias. The dual nature of this process aligns with recent theoretical discussions emphasizing both the adaptive and maladaptive consequences of prolonged AI interaction [10].

Algorithmic habituation should be distinguished from adjacent constructs such as automation bias, cognitive offloading, and technology acceptance. While automation bias refers to a decision-level tendency to favor algorithmic outputs over human judgment, and cognitive offloading describes the delegation of cognitive tasks to external systems, algorithmic habituation captures a broader, processual phenomenon involving the gradual stabilization of cognitive, behavioral, and evaluative patterns through repeated interaction. Unlike technology acceptance models, which focus on attitudes and adoption, algorithmic habituation concerns the transformation of cognition itself over time. Thus, the construct is not reducible to these frameworks but integrates and extends them within a dynamic, systems-level perspective.

### 1.4. Aims and Contributions of the Study

Building on the theoretical and empirical gaps identified above, the present paper aims to develop a comprehensive framework for understanding algorithmic habituation within human–AI interaction. Specifically, the study seeks to:

(1) Conceptualize algorithmic habituation as a distinct cognitive process grounded in co-adaptation and neurocognitive theories;

(2) Integrate perspectives from habituation, neuroplasticity, predictive processing, and systems theory into a unified explanatory model;

(3) Propose a mechanistic account of how repeated interaction with AI systems leads to cognitive stabilization and behavioral standardization;

(4) Develop a typology of algorithmic habituation across cognitive, decisional, creative, and moral domains;

(5) Outline empirical pathways for future research, including measurement, experimental validation, and longitudinal analysis.

By introducing and formalizing the concept of algorithmic habituation, this paper contributes to a deeper understanding of how AI systems reshape human cognition over time. In doing so, it advances current debates on digital wellbeing, AI ethics, and the future of human–AI interaction, positioning user adaptation as a central phenomenon in the study of intelligent systems.

To enhance conceptual clarity, it is important to note that the present framework integrates both literature-based syntheses and original theoretical extensions. Specifically, while several mechanisms discussed (e.g., cognitive offloading, predictive processing, and automation bias) are grounded in existing research, constructs such as “algorithmic habituation,” “behavioral compression,” and the proposed typology are introduced here as original conceptual contributions. Throughout the manuscript, we explicitly distinguish between empirically supported findings and theoretically proposed extensions where necessary.

## 2. Theoretical Foundations

### 2.1. Habituation in Classical Psychology and Neuroscience

Habituation represents one of the most fundamental forms of learning, traditionally defined as a progressive decrease in response to repeated, non-threatening stimuli. Its conceptual roots span both psychology and neuroscience, where it has been studied as a basic adaptive mechanism underlying behavioral efficiency and cognitive filtering. Early historical accounts emphasize habituation as a core process through which organisms optimize responses to environmental regularities, minimizing unnecessary cognitive and physiological expenditure [13].

From a neurobiological and psychological perspective, habituation has been conceptualized as a dynamic process involving both sensory attenuation and higher-order cognitive modulation [14]. Rather than a simple decline in responsiveness, habituation reflects complex interactions between attention, memory, and expectation, contributing to the selective prioritization of relevant stimuli [15]. Contemporary frameworks further highlight that habituation plays a critical role in maintaining cognitive stability, allowing individuals to operate efficiently in environments characterized by repeated patterns and predictable inputs [15,16].

Neurophysiological studies have demonstrated that habituation involves both synaptic and network-level changes, reflecting adaptive modifications in neural processing pathways [17]. Comparative research across developmental and animal models suggests that habituation operates as a generalizable mechanism, bridging basic neural processes and higher-order cognitive functions [18]. This integrative perspective positions habituation as a foundational process that supports learning, perception, and behavioral regulation across contexts [19].

Moreover, theoretical developments such as the dual-process theory propose that habituation interacts with sensitization processes, resulting in a dynamic balance between decreased and increased responsiveness depending on contextual factors [20]. This interaction underscores the adaptive nature of habituation, which is not merely suppressive but context-sensitive and functionally flexible. In associative learning frameworks, habituation has also been linked to changes in stimulus salience, influencing how organisms encode and respond to environmental cues over time [21].

Recent research has extended the concept of habituation into attentional domains, demonstrating that repeated exposure to stimuli can reduce attentional capture, thereby shaping perceptual and cognitive prioritization mechanisms [22]. Taken together, these perspectives converge on a critical insight: habituation is not a passive decline in responsiveness but an active process of cognitive optimization, enabling individuals to adapt efficiently to structured and repetitive environments.

### 2.2. Neuroplasticity and Adaptive Reorganization

The mechanisms underlying habituation are deeply intertwined with processes of neuroplasticity, defined as the brain’s capacity to reorganize its structure and function in response to experience. Neuroplasticity provides the biological substrate through which repeated interactions lead to enduring cognitive and behavioral changes.

Contemporary research emphasizes that the brain is not a static system but a dynamic and continuously evolving network, capable of adapting to both internal and external demands [23]. This adaptability is essential for learning, recovery, and cognitive flexibility, allowing individuals to refine neural representations in response to environmental regularities [24]. In clinical and applied contexts, neuroplasticity has been shown to support functional recovery following neurological disruption, further underscoring its role in adaptive reorganization [25].

Recent advances highlight that neuroplasticity is not limited to early development but remains active throughout the lifespan, enabling continuous adaptation to novel environments and technologies [26]. This has significant implications for understanding how repeated interaction with AI systems may induce long-term cognitive changes, as neural circuits become tuned to the regularities and constraints of algorithmic environments.

Theoretical perspectives on neural reuse and cognitive adaptability further suggest that existing neural structures can be repurposed to support new forms of cognition, including those mediated by technological interaction [27]. This aligns with emerging views that cognition is not confined to the brain but extends across distributed systems involving tools, environments, and interactive agents.

More recent contributions reinforce the idea that neuroplasticity underpins adaptive processes across domains, including perception, motor control, and higher-order cognition [28,29]. Within this framework, repeated interaction with structured stimuli—such as AI-generated outputs—can lead to the stabilization of neural and cognitive patterns, reflecting a form of experience-dependent reorganization.

Thus, neuroplasticity provides a critical bridge between classical habituation and contemporary human–AI interaction. It explains how repeated exposure to algorithmic patterns can lead not only to behavioral adjustments but also to deeper cognitive restructuring, supporting the emergence of stable interaction routines over time.

### 2.3. Predictive Processing and Expectation Formation

While habituation and neuroplasticity explain the adaptive consequences of repeated exposure, the predictive processing framework offers a mechanistic account of how these processes are organized within the brain. Predictive processing posits that cognition operates as a hierarchical system of prediction generation and error minimization, where the brain continuously anticipates incoming sensory information and updates its internal models based on prediction errors [30].

Empirical evidence from electrophysiological studies indicates that predictive mechanisms are engaged early in perceptual processing, particularly in response to auditory and sensory regularities [31]. These findings suggest that the brain actively constructs expectations about the environment, using prior experience to guide perception and action. From this perspective, repeated exposure to consistent patterns leads to stronger predictions and reduced processing demands, as expected inputs generate minimal prediction error.

Theoretical developments further elaborate that predictive processing extends beyond perception to encompass cognition, action, and conscious experience [32]. Within this framework, representations are not static but dynamically constructed through ongoing interactions between top-down predictions and bottom-up signals [33]. This dynamic interplay allows the system to optimize efficiency by reducing uncertainty and stabilizing expectations.

Recent work emphasizes that predictive processing is inherently action-oriented, involving the anticipation of both sensory and behavioral outcomes [34]. This has direct relevance for human–AI interaction, where users engage in goal-directed activities mediated by predictive systems. Over time, users may internalize the regularities of AI outputs, forming expectations that guide both interpretation and decision-making.

Importantly, predictive processing entails both benefits and costs. While it enhances efficiency and facilitates rapid adaptation, it may also lead to reduced sensitivity to novel or unexpected information, particularly when predictions become overly stable [35]. Experimental and phenomenological studies further demonstrate that expectations can shape perceptual experience itself, highlighting the deep integration of prediction and cognition [36].

Within the context of human–AI interaction, predictive processing provides a compelling framework for understanding how users come to anticipate and rely on algorithmic outputs. Repeated exposure to consistent AI responses reduces prediction error, reinforcing expectations and stabilizing interaction patterns. This process represents a key mechanism underlying the emergence of algorithmic habituation, where cognitive systems adapt to the statistical regularities of AI environments.

### 2.4. Toward an Integrated Framework: From Habituation to Algorithmic Habituation

Building on the perspectives outlined above, it becomes possible to conceptualize algorithmic habituation as the convergence of three core processes: habituation as behavioral and cognitive optimization, neuroplasticity as adaptive neural reorganization, and predictive processing as expectation-driven cognition.

Recent integrative approaches in cognitive science and systems theory emphasize that cognition emerges from entangled, recursive interactions between agents and their environments [37]. Within such frameworks, human–AI interaction can be understood as a coupled system in which both user and technology participate in continuous co-adaptation. This perspective aligns with the notion of autopoietic systems, where stability and change emerge through ongoing feedback loops rather than linear causation.

At the same time, critical perspectives on AI-mediated cognition highlight the risk of constructing illusory measurements and synthetic constructs, particularly when complex human processes are reduced to algorithmic outputs [38]. This underscores the importance of carefully theorizing the mechanisms through which AI systems influence cognition, avoiding simplistic or reductionist interpretations.

In this context, algorithmic habituation can be defined as a system-level phenomenon, emerging from the interaction between neural adaptation, predictive modeling, and repeated exposure to structured algorithmic outputs. It represents a shift from stimulus-driven habituation to interaction-driven habituation, where the object of adaptation is not a static stimulus but a dynamic, responsive system.

This integrated framework provides the theoretical foundation for understanding how human cognition evolves in AI-rich environments. By linking classical learning mechanisms with contemporary models of cognition and systems interaction, it establishes a coherent basis for analyzing the processes through which users become progressively attuned to algorithmic structures—ultimately setting the stage for the formal modeling of algorithmic habituation in the following sections.

Importantly, the transition from neural adaptation to higher-order cognitive and socio-behavioral effects should be understood as indirect and multi-layered rather than strictly linear. While neuroplastic mechanisms provide the substrate for adaptation, their expression at the level of decision-making, creativity, and moral judgment is mediated by cognitive, contextual, and social factors. This distinction highlights that algorithmic habituation operates across levels of organization, without assuming a one-to-one correspondence between neural and behavioral change.

## 3. Mechanism of Algorithmic Habituation

### 3.1. The Human–AI Co-Adaptation Loop

Building upon the theoretical foundations outlined above, algorithmic habituation can be understood as an emergent property of human–AI co-adaptive systems, in which both agents dynamically adjust to each other through continuous interaction. Contemporary perspectives on human–AI collaboration emphasize that such systems are no longer linear or tool-based but function as reciprocal adaptive ecosystems, characterized by feedback loops, shared representations, and evolving interaction patterns [39].

In these systems, AI does not merely respond to user input but actively shapes the interaction space through predictive outputs and adaptive responses. At the same time, users progressively modify their behavior, expectations, and decision-making strategies to align with system regularities. This bidirectional process reflects a co-evolutionary dynamic, where human cognition and algorithmic structures become increasingly entangled over time [40].

The human-in-the-loop paradigm further reinforces this perspective, highlighting that effective interaction depends on continuous feedback integration between human and system, rather than isolated decision-making processes [41]. In this context, co-adaptation is not an auxiliary feature but a defining characteristic of intelligent systems, particularly in domains involving generative AI and adaptive interfaces.

Recent advances in human–robot interaction and symbiotic AI systems suggest that co-adaptation can reach a level of synergistic alignment, where both agents optimize their behavior through reciprocal feedback mechanisms [42,43]. This alignment is further extended at the societal and organizational level, where AI systems contribute to the co-evolution of knowledge structures and decision-making processes [44]. Together, these developments establish the human–AI interaction loop as a central mechanism through which algorithmic habituation emerges.

### 3.2. Repetition and Pattern Extraction

At the core of the co-adaptation loop lies repetition, which enables the extraction of patterns from repeated interactions. Through continuous exposure to AI-generated outputs, users begin to identify regularities in response structures, linguistic formulations, and decision pathways.

The temporal dynamics of this process remain an important consideration. Algorithmic habituation may emerge across varying timescales, depending on both the frequency and intensity of interaction with AI systems. Short-term exposure may produce initial pattern recognition and expectation formation, whereas sustained and repeated engagement is more likely to result in stabilized cognitive routines and behavioral convergence. The distinction between interaction frequency and depth suggests that habituation is not solely a function of time, but of cumulative exposure and cognitive reliance on system outputs.

From a cognitive perspective, repeated interaction facilitates pattern detection, allowing users to anticipate system behavior with increasing accuracy. This process reduces uncertainty and enhances interaction efficiency, as users learn to navigate algorithmic structures more effectively. At the same time, AI systems refine their outputs based on user input, reinforcing stable interaction patterns.

In adaptive systems theory, this process is described as iterative convergence, where both agents progressively reduce variability in interaction through repeated feedback cycles [40]. As a result, interaction becomes more predictable, structured, and efficient, laying the groundwork for the stabilization of expectations and behaviors.

### 3.3. Expectation Stabilization and Cognitive Economy

As patterns become internalized, users develop stable expectations regarding AI responses. Within a predictive processing framework, this corresponds to the reduction in prediction error, as expected outputs align with internal models. Over time, this leads to a state of expectation stabilization, where interaction with AI systems becomes increasingly automatic and less cognitively demanding.

This process gives rise to cognitive economy, defined as the optimization of mental effort through reliance on established patterns and heuristics. By leveraging predictable AI outputs, users can reduce the need for active problem-solving, thereby conserving cognitive resources. While this enhances efficiency, it also introduces the risk of over-reliance on algorithmic structures.

In human-in-the-loop optimization studies, such efficiency gains are often framed as desirable outcomes, reflecting improved system usability and performance [42]. However, from a cognitive perspective, this shift also signals a transition from active engagement to pattern-based interaction, where decisions are increasingly guided by learned expectations rather than deliberate reasoning.

### 3.4. Reduction in Reflexivity and Behavioral Compression

A critical consequence of expectation stabilization is the reduction in reflexivity, understood as the diminished tendency to critically evaluate or question system outputs. As interaction becomes more predictable, users may rely on automated responses rather than engaging in reflective reasoning processes.

This leads to what we conceptualize as behavioral compression, a process in which diverse cognitive and behavioral responses are reduced to a narrower set of standardized patterns. While related to existing notions of behavioral convergence in adaptive systems, this construct is proposed here as an extension of the algorithmic habituation framework. In the context of AI interaction, this manifests as convergence toward similar problem-solving strategies, linguistic structures, and decision pathways.

From a systems perspective, such compression reflects the stabilization of interaction dynamics within a constrained solution space, where variability is minimized in favor of efficiency and predictability [39]. While this can enhance performance in routine tasks, it may also limit creativity, critical thinking, and adaptive flexibility.

Moreover, the increasing integration of agentic AI systems into organizational and social contexts suggests that these effects may scale beyond individual cognition, influencing collective behaviors and knowledge structures [44]. Thus, the reduction in reflexivity is not merely an individual phenomenon but part of a broader transformation in human–AI ecosystems.

### 3.5. Formal Model

**Figure 1:**
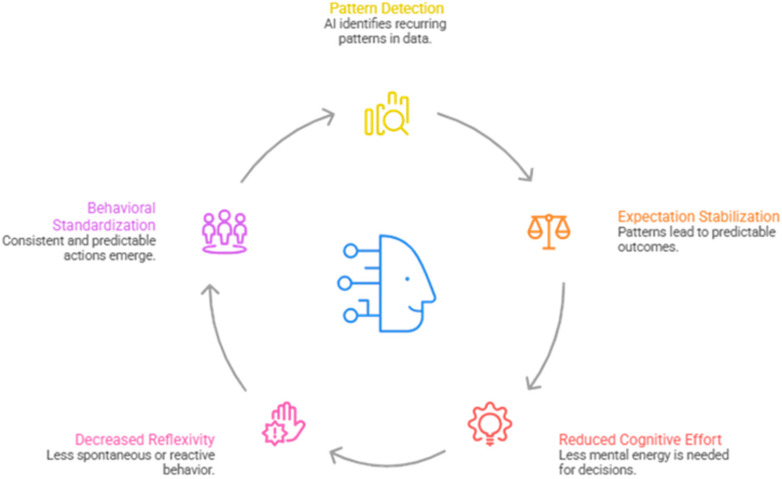
Conceptual model of algorithmic habituation.

The mechanism of algorithmic habituation can be conceptually synthesized into a recursive loop model, as illustrated in Figure 1.

The model captures the sequential and recursive dynamics of human–AI interaction:

1. Pattern detection—AI identifies recurring structures in user input and interaction data.

2. Expectation stabilization—users form stable expectations based on predictable AI responses.

3. Reduced cognitive effort—reliance on established patterns decreases the need for active processing.

4. Decreased reflexivity—critical evaluation diminishes as interaction becomes automated.

5. Behavioral standardization—responses converge toward efficient but constrained patterns.

Although the model is presented conceptually, it can be expressed in simplified functional terms. Let *H_t_* represent the level of habituation at time *t*, and *I_t_* the intensity of interaction with AI systems. Then, habituation can be described as an iterative function:

*H*_*t*+1_ = *H*_*t*_ + *α* × *f* (*I_t_,E_t_,P_t_*)

where *E_t_* represents expectation stabilization and *P_t_* pattern regularity, and α is a learning rate parameter. This formulation is not intended as a fully specified computational model but as a heuristic representation that illustrates the recursive and accumulative nature of algorithmic habituation, supporting its future operationalization.

From a theoretical standpoint, these stages can be interpreted as a series of propositions that describe the expected progression of algorithmic habituation. Specifically, repeated interaction with AI systems is expected to increase pattern detection, which in turn stabilizes expectations, reduces cognitive effort, decreases reflexivity, and ultimately leads to behavioral standardization. These propositions provide a foundation for future empirical testing using experimental and longitudinal designs.

These stages form a closed feedback loop, where each cycle reinforces the next, leading to progressively stronger habituation effects. Importantly, the loop is not strictly linear but recursive, allowing for continuous reinforcement and potential amplification over time.

Within this framework, algorithmic habituation emerges as a system-level attractor state, characterized by stability, efficiency, and reduced variability in human–AI interaction. This formulation aligns with broader models of adaptive systems, where repeated feedback leads to the emergence of stable behavioral patterns [40].

At the same time, the model highlights the dual nature of algorithmic habituation: while it supports cognitive efficiency and streamlined interaction, it also raises critical concerns regarding reduced reflexivity, automation bias, and the standardization of cognition. These implications are further explored in the following sections.

To enhance the empirical tractability of the model, each stage of the loop can be associated with observable indicators. For example, pattern detection may be reflected in reduced variability of user inputs, expectation stabilization in decreased response latency, reduced cognitive effort in shorter interaction cycles, and decreased reflexivity in lower rates of critical revision or override of AI outputs. These indicators provide a preliminary bridge between the conceptual framework and measurable behavioral phenomena.

From a predictive perspective, the model suggests that higher interaction frequency and predictability of system outputs will be associated with accelerated habituation, whereas variability, uncertainty, or conflicting outputs may disrupt or slow the stabilization process. These assumptions provide a basis for future empirical testing of the model’s dynamic properties.

### 3.6. Moderating Factors and Individual Differences

The trajectory and intensity of algorithmic habituation are unlikely to be uniform across users. Individual differences such as AI literacy, domain expertise, cognitive style, and task complexity may significantly moderate the co-adaptation loop. For instance, users with higher AI literacy may maintain greater reflexivity and resist premature expectation stabilization, while novice users may enter habituation cycles more rapidly due to reliance on system outputs. Similarly, complex or uncertain tasks may slow habituation by preserving cognitive engagement, whereas routine tasks may accelerate it. These moderating factors suggest that algorithmic habituation should be understood not as a fixed process but as a context-sensitive dynamic shaped by both user characteristics and interaction environments.

These factors may also function as boundary conditions, determining not only the intensity but also the qualitative form of habituation that emerges.

## 4. Typology of Algorithmic Habituation

**Figure 2:**
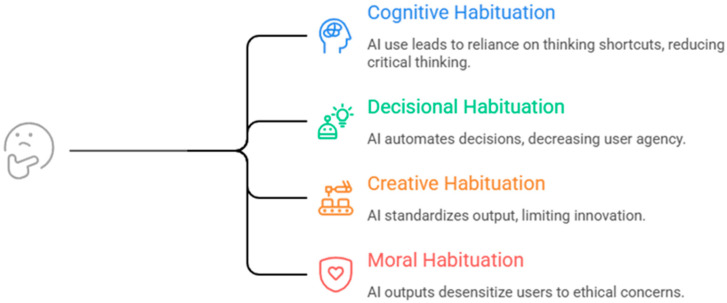
Typology of algorithmic habituation.

Algorithmic habituation does not manifest as a uniform cognitive process but unfolds across multiple domains of human functioning. Building on the mechanism described in the previous section, this paper proposes a four-dimensional typology of algorithmic habituation, capturing distinct yet interrelated forms through which repeated interaction with AI systems reshapes cognition, decision-making, creativity, and moral evaluation (Figure 2).

In Figure 2, each quadrant represents a distinct domain: cognitive (thinking shortcuts), decisional (automation of decisions), creative (standardization of outputs), and moral (ethical desensitization), illustrated with domain-specific symbolic indicators.

### 4.1. Cognitive Habituation (Thinking Shortcuts)

Cognitive habituation refers to the gradual reliance on simplified cognitive strategies and heuristics as a result of repeated interaction with AI systems. As users become familiar with predictable response patterns, they increasingly adopt thinking shortcuts, reducing the need for analytical reasoning and critical evaluation.

This process can be understood in relation to the Einstellung effect, where prior experience with a particular solution constrains the exploration of alternative approaches [45,46]. In AI-mediated environments, repeated exposure to structured outputs may reinforce fixed cognitive pathways, limiting flexibility and promoting habitual thinking patterns.

While such shortcuts enhance efficiency, they may also contribute to cognitive narrowing, where users preferentially rely on familiar algorithmic structures rather than engaging in deeper problem-solving processes. This aligns with broader perspectives suggesting that ignoring complexity can be perceived as cognitively advantageous, particularly in decision contexts characterized by information overload [47].

Thus, cognitive habituation represents a shift from deliberative cognition to pattern-based processing, where efficiency is prioritized over critical engagement.

### 4.2. Decisional Habituation (Automation of Decisions)

While cognitive habituation and decisional habituation are closely related, they operate at distinct levels of processing. Cognitive habituation refers to the internal simplification of thinking strategies and the increasing reliance on heuristics, whereas decisional habituation reflects the externalization of these processes into action, where choices are guided or delegated to AI systems. In this sense, cognitive habituation precedes and enables decisional habituation, but the latter involves a shift from internal cognitive economy to observable decision behavior.

Decisional habituation emerges when users increasingly rely on AI systems to guide or automate decision-making processes. Over time, repeated exposure to algorithmic recommendations leads to the internalization of decision pathways, reducing the perceived need for independent judgment.

Research on human–automation interaction indicates that decision-makers and automated systems are often highly compatible, with users readily integrating algorithmic suggestions into their choices [48]. This dynamic can enhance performance in structured environments but may also foster over-reliance, particularly when users begin to trust system outputs without sufficient scrutiny.

Experimental studies in decision-making contexts demonstrate that choices can be shaped by task structure and environmental constraints, often leading to rapid, heuristic-based responses [49,50]. In AI-mediated environments, such tendencies are amplified, as systems provide ready-made solutions that reduce cognitive effort.

Decisional habituation thus reflects a transition from active decision-making to assisted or delegated cognition, where agency is partially transferred to algorithmic systems. While this can improve efficiency, it raises critical concerns regarding autonomy, accountability, and the erosion of human control.

This distinction ensures that decisional habituation is not reduced to cognitive simplification alone, but reflects a shift in the locus of control from internal reasoning to externally guided action.

### 4.3. Creative Habituation (Standardization of Output)

Creative habituation refers to the progressive standardization of creative processes and outputs resulting from repeated interaction with generative AI systems. As users adapt to the stylistic and structural regularities of AI-generated content, their own creative production may converge toward similar patterns.

In this context, creative habituation is conceptualized as an extension of existing findings on idea convergence and homogenization, integrating them into the broader mechanism of algorithmic habituation proposed in this study.

Recent research highlights the homogenization effect of large language models, showing that exposure to AI-generated ideas can reduce diversity in human creative ideation [51]. This occurs as users internalize dominant patterns and replicate them in subsequent tasks, leading to reduced originality and variability.

While AI systems can enhance creativity by providing inspiration and expanding idea spaces, they also introduce constraints through their inherent biases and training data distributions. Over time, this may lead to creative convergence, where outputs become increasingly uniform and predictable.

Creative habituation thus reflects a paradox: while AI tools expand creative possibilities, they may simultaneously narrow the space of exploration, reinforcing dominant patterns and limiting innovation.

### 4.4. Moral Habituation (Ethical Desensitization to AI Outputs)

Moral habituation represents one of the most critical and underexplored dimensions of algorithmic habituation. It refers to the progressive desensitization to ethical concerns associated with AI outputs, resulting from repeated exposure and normalization of algorithmic decision-making.

In high-stakes contexts, such as military or policy decision-making, concerns have been raised regarding the potential erosion of human moral agency in AI-mediated environments [52]. As users become accustomed to delegating decisions to AI systems, the threshold for ethical scrutiny may gradually decrease.

The phenomenon of ethical leniency through repeated exposure has been described as a gradual process, where individuals become increasingly tolerant of questionable practices over time [53]. In the context of AI, this may manifest as reduced sensitivity to biases, inaccuracies, or ethical implications embedded in algorithmic outputs.

At the same time, technological familiarity plays a complex role, influencing both trust in AI systems and perceptions of risk [54]. Users who are more accustomed to AI may exhibit lower levels of concern, further reinforcing habituation effects.

Insights from digital environments, including gaming and simulated systems, suggest that repeated interaction with artificial agents can reshape moral reasoning and ethical judgment [55]. Complementary research on moral desensitization indicates that exposure to digital content can reduce emotional responsiveness to ethical issues, particularly when mediated by technology [56].

Moral habituation thus represents a critical boundary condition for algorithmic habituation, highlighting the potential risks associated with the normalization of AI-mediated decision-making. Unlike other forms, it directly impacts ethical reasoning, responsibility, and the preservation of human agency.

The four dimensions—cognitive, decisional, creative, and moral—illustrate that algorithmic habituation operates across multiple layers of human functioning. These dimensions are not independent but interact dynamically, reinforcing each other within the broader co-adaptive loop.

The typology underscores that algorithmic habituation is not merely a cognitive phenomenon but a multidimensional transformation, affecting how individuals think, decide, create, and evaluate ethical implications in AI-rich environments. This comprehensive perspective provides a foundation for both theoretical refinement and empirical investigation, as well as for addressing the broader societal implications of human–AI interaction.

From a moral psychology perspective, this process may also be interpreted through mechanisms such as moral disengagement, normalization of deviance, and gradual threshold shifting in ethical evaluation. Repeated exposure to algorithmic decisions may reduce emotional salience and moral scrutiny, particularly when outcomes are framed as system-generated rather than agent-driven. This aligns with broader findings in moral cognition suggesting that repeated exposure to ethically ambiguous situations can recalibrate normative judgments over time.

Importantly, these dimensions should not be understood as isolated categories but as interdependent processes within the broader habituation loop. Cognitive habituation may facilitate decisional automation, while repeated decisional reliance may, in turn, reinforce cognitive shortcuts. Similarly, creative and moral habituation may emerge as downstream effects of stabilized interaction patterns. This interdependence suggests that algorithmic habituation operates as a layered and mutually reinforcing system rather than as a set of discrete phenomena.

## 5. Consequences and Risks

Building directly on the mechanism described in the previous section, the emergence of algorithmic habituation entails a complex set of consequences that extend across cognitive, behavioral, and socio-ethical domains. While the mechanisms described in previous sections highlight adaptive benefits such as efficiency and cognitive economy, these processes also introduce significant risks related to diminished critical engagement, over-reliance on automation, and the transformation of identity and agency. This section synthesizes these implications into four major domains.

### 5.1. Cognitive Offloading and Reduced Critical Thinking

One of the most immediate consequences of algorithmic habituation is the increasing reliance on cognitive offloading, defined as the delegation of cognitive processes to external systems. In the context of AI, this involves outsourcing memory, reasoning, and problem-solving tasks to algorithmic agents.

At the same time, cognitive offloading may also enable higher-order cognitive functioning under certain conditions. By reducing the burden of routine processing, AI systems can free cognitive resources for integrative reasoning, problem framing, and creative exploration. This suggests that the impact of algorithmic habituation is not uniformly negative, but depends on how offloading is integrated within broader cognitive strategies.

Recent research suggests that the widespread use of AI tools may significantly impact critical thinking abilities, as users rely on system outputs rather than engaging in independent analysis [57]. Cognitive offloading is not inherently detrimental; it can enhance efficiency and reduce cognitive load. However, its long-term effects depend on how it reshapes cognitive engagement and learning processes.

Empirical evidence indicates that both the actual and perceived reliability of tools influence the extent to which individuals offload cognitive tasks [58]. As AI systems become more accurate and trustworthy, users may increasingly defer to them, reinforcing habituation effects. In educational contexts, this dynamic raises concerns about the erosion of analytical skills, particularly when offloading replaces rather than complements cognitive effort [59].

The paradox of AI-supported cognition lies in the tension between efficiency and critical engagement. While reduced cognitive load can facilitate performance, it may also lead to diminished reflective thinking and reduced intellectual autonomy [60,61]. At the same time, emerging approaches in AI-assisted learning attempt to balance offloading with interactive engagement, suggesting that the impact of AI on cognition is not fixed but context-dependent [62].

### 5.2. Automation Bias and Over-Reliance

Closely related to cognitive offloading is the phenomenon of automation bias, defined as the tendency to favor automated decisions over human judgment, even when the system may be incorrect. Within the framework of algorithmic habituation, automation bias represents a natural progression of repeated reliance on AI systems.

In high-stakes domains such as healthcare, automation bias has been identified as a critical risk, potentially leading to errors when users fail to question or override system outputs [63]. This issue has long been recognized in the context of decision support systems, where users may overtrust automated recommendations [64].

Recent reviews emphasize that automation bias remains a central challenge in human–AI collaboration, particularly as systems become more complex and opaque [65]. Behavioral studies further indicate that reliance on AI can alter diagnostic and decision-making processes, potentially reducing skill retention and independent reasoning over time [66].

Trust plays a crucial role in shaping these dynamics. Foundational research on trust in automation highlights that both overtrust and undertrust can impair performance, underscoring the need for calibrated reliance on AI systems [67]. Within algorithmic habituation, repeated positive interactions may gradually increase trust, reinforcing over-reliance and reducing critical evaluation.

Thus, automation bias can be understood as a behavioral manifestation of algorithmic habituation, where repeated exposure to reliable outputs leads to diminished vigilance and increased dependence on automated systems.

### 5.3. Standardization of Cognition and Behavior

Another significant consequence of algorithmic habituation is the standardization of cognition and behavior, reflecting the convergence of human responses toward algorithmically structured patterns. As users adapt to AI systems, their cognitive processes may become increasingly aligned with the logic, constraints, and biases embedded in these systems.

From a computational perspective, models of cognition and artificial intelligence suggest that human and machine processes can be integrated within shared frameworks, leading to overlapping patterns of reasoning and decision-making [68,69]. While this convergence can enhance interoperability, it also raises concerns regarding the loss of diversity in thought and behavior.

Standardization has been identified as a key challenge in the development and deployment of AI technologies, particularly in ensuring consistency and reliability across systems [70,71]. However, when applied to human cognition, standardization may have unintended consequences, including reduced variability, creativity, and adaptability.

Recent discussions emphasize that the increasing influence of AI systems may contribute to broader processes of cognitive and social standardization, shaping how individuals think, communicate, and interact [72]. At the same time, tensions between standardization and customization highlight the complex dynamics of AI-mediated environments, where users seek personalized experiences within structurally constrained systems [73].

Efforts toward international standardization of AI technologies further reinforce these trends, promoting uniformity in design and implementation while potentially amplifying their influence on human cognition [74]. Within this context, algorithmic habituation can be seen as a driver of cognitive convergence, where repeated interaction with standardized systems leads to increasingly homogeneous patterns of behavior.

### 5.4. Implications for Identity and Agency

Perhaps the most profound consequences of algorithmic habituation relate to its impact on identity and agency. As AI systems become integrated into cognitive processes, they begin to influence not only what individuals do but also how they perceive themselves as agents.

Recent theoretical work introduces the concept of the algorithmic self, suggesting that AI systems contribute to the construction of identity [75]. Building on this perspective, we extend this argument by proposing that such identity-related processes may be partially mediated by mechanisms of algorithmic habituation.

Research on AI and identity further emphasizes the complex relationships between creators, users, and algorithmic outputs, raising questions about authorship, ownership, and responsibility [76]. At the same time, emerging analyses point to potential threats to human agency, particularly when decision-making is increasingly mediated by AI systems [77].

Philosophical and ethical perspectives underscore that agency involves not only the capacity to act but also the ability to reflect, evaluate, and take responsibility for decisions [78]. Within the context of algorithmic habituation, the reduction in reflexivity and the delegation of decision-making may undermine these capacities, leading to a reconfiguration of agency.

Empirical research on digital agency suggests that the impact of AI on autonomy is not uniform, with individuals experiencing both empowerment and constraint depending on context and usage patterns [79]. Similarly, the increasing use of personalized AI agents highlights their role in shaping self-presentation, decision-making, and identity management [80].

Ultimately, algorithmic habituation raises fundamental questions about the future of human agency in AI-rich environments. As users become progressively attuned to algorithmic systems, the boundaries between human intention and algorithmic influence become increasingly blurred, suggesting the emergence of hybrid forms of cognition and identity.

The consequences outlined above reveal that algorithmic habituation is not merely a cognitive adaptation but a multilevel transformation affecting thinking, decision-making, behavior, and identity. While it offers significant benefits in terms of efficiency and usability, it also introduces critical risks that must be carefully managed.

Understanding these risks is essential for developing responsible AI systems and fostering digital environments that support not only performance but also critical thinking, autonomy, and ethical awareness. These considerations provide the foundation for the next section, which outlines empirical pathways for investigating and operationalizing algorithmic habituation.

These considerations also have implications for the design of human-centered AI systems. Introducing variability, transparency, and opportunities for user reflection may help mitigate excessive habituation, preserving critical engagement and adaptive flexibility within human–AI interaction.

## 6. Conclusions

The present study introduces algorithmic habituation as a framework for understanding how repeated interaction with artificial intelligence systems reshapes human cognition, behavior, and agency. Importantly, while the framework builds on established theories from cognitive science and human–AI interaction, its primary contribution lies in the formalization of algorithmic habituation as a distinct, integrative construct.

Furthermore, the proposed typology—encompassing cognitive, decisional, creative, and moral habituation—offers a structured framework for analyzing the differentiated impacts of AI systems across domains of human functioning.

The findings highlight that algorithmic habituation is not inherently detrimental but involves a dual dynamic. On the one hand, it supports efficiency, reduces cognitive load, and enables smoother interaction with increasingly complex systems. On the other hand, it introduces risks related to diminished critical thinking, automation bias, standardization of cognition, and the potential erosion of human agency.

Future research should focus on the empirical operationalization of algorithmic habituation. This includes the development of validated measurement instruments, experimental paradigms capable of capturing short-term adaptation processes, and longitudinal designs that track the evolution of habituation over time. Additionally, integrating neurocognitive methods such as EEG or behavioral tracking could provide deeper insight into the underlying mechanisms.

Another important direction concerns the contextual variability of algorithmic habituation. The extent and consequences of habituation are likely to differ across domains such as education, healthcare, creative industries, and decision-making environments. Understanding these contextual differences is essential for designing AI systems that support rather than constrain human cognition.

This study has several limitations that should be acknowledged. First, the proposed framework is conceptual in nature and has not yet been empirically validated. While the paper outlines potential methodological pathways, including experimental and longitudinal designs, the mechanisms described here should be interpreted as theoretically grounded propositions requiring systematic empirical testing. Second, the generalizability of algorithmic habituation is likely to vary across domains, interaction contexts, and user populations, particularly given differences in task structure and levels of exposure to AI systems. Third, although the framework acknowledges the potential role of individual differences, it does not explicitly model how factors such as AI literacy, domain expertise, or cognitive style may moderate the habituation process. Future research should therefore investigate these variables as boundary conditions shaping the trajectory and intensity of algorithmic habituation.

Rather than reiterating prior arguments, the present framework invites a shift toward investigating how human cognition evolves within adaptive technological environments. The central contribution lies not only in defining algorithmic habituation but in positioning it as a testable, system-level construct with implications for the design of human-centered AI systems.

### Empirical Operationalization and Measurement Directions

To extend the directions outlined above, the following section provides a more detailed operational framework.

Future research should prioritize the operationalization of algorithmic habituation through multi-method approaches. Psychometric scale development could target dimensions such as reliance on AI outputs, reduction in cognitive effort, and perceived predictability of systems. Experimental paradigms may compare repeated versus novel interaction conditions to capture short-term adaptation dynamics. Longitudinal designs are particularly suited to examining the cumulative effects of interaction frequency and duration. Behavioral indicators, including response latency, variability of outputs, and resistance to contradictory information, may provide indirect measures of habituation. Additionally, neurocognitive methods such as EEG could be used to assess changes in attentional engagement and expectation processing over repeated AI interactions.

## Data Availability

No new data were created or analyzed in this study. Data sharing is not applicable to this article.
